# Rationale and Design of the Hamburg City Health Study

**DOI:** 10.1007/s10654-019-00577-4

**Published:** 2019-11-08

**Authors:** Annika Jagodzinski, Christoffer Johansen, Uwe Koch-Gromus, Ghazal Aarabi, Gerhard Adam, Sven Anders, Matthias Augustin, Ramona B. der Kellen, Thomas Beikler, Christian-Alexander Behrendt, Christian S. Betz, Carsten Bokemeyer, Katrin Borof, Peer Briken, Chia-Jung Busch, Christian Büchel, Stefanie Brassen, Eike S. Debus, Larissa Eggers, Jens Fiehler, Jürgen Gallinat, Simone Gellißen, Christian Gerloff, Evaldas Girdauskas, Martin Gosau, Markus Graefen, Martin Härter, Volker Harth, Christoph Heidemann, Guido Heydecke, Tobias B. Huber, Yassin Hussein, Marvin O. Kampf, Olaf von dem Knesebeck, Alexander Konnopka, Hans-Helmut König, Robert Kromer, Christian Kubisch, Simone Kühn, Sonja Loges, Bernd Löwe, Gunnar Lund, Christian Meyer, Lina Nagel, Albert Nienhaus, Klaus Pantel, Elina Petersen, Klaus Püschel, Hermann Reichenspurner, Guido Sauter, Martin Scherer, Katharina Scherschel, Ulrich Schiffner, Renate B. Schnabel, Holger Schulz, Ralf Smeets, Vladislavs Sokalskis, Martin S. Spitzer, Claudia Terschüren, Imke Thederan, Tom Thoma, Götz Thomalla, Benjamin Waschki, Karl Wegscheider, Jan-Per Wenzel, Susanne Wiese, Birgit-Christiane Zyriax, Tanja Zeller, Stefan Blankenberg

**Affiliations:** 1 Department of General and Interventional Cardiology, University Heart and Vascular Center, Hamburg, Germany; 2 German Center for Cardiovascular Research (DZHK) Partner Site Hamburg/Lübeck/Kiel, Munich, Germany; 3 Epidemiological Study Center, University Medical Center Hamburg-Eppendorf (UKE), Hamburg, Germany; 4 Oncology Clinic, Finsen Center, Copenhagen, Denmark; 5 Survivorship Research Unit, The Danish Cancer Society Research Center, Copenhagen, Denmark; 6 Institute for Medical Biometry and Epidemiology (IMBE), University Medical Center Hamburg-Eppendorf (UKE), Hamburg, Germany; 7 Faculty of Medicine, University Medical Center Hamburg-Eppendorf (UKE), Hamburg, Germany; 8 Department of Prosthetic Dentistry, Center for Dental and Oral Medicine, University Medical Center Hamburg-Eppendorf, Hamburg, Germany; 9 Department of Diagnostics and Interventional Radiology and Nuclear Medicine, University Medical Center Hamburg-Eppendorf (UKE), Hamburg, Germany; 10 Department for Forensic Medicine, University Medical Center Hamburg-Eppendorf (UKE), Hamburg, Germany; 11 Institute for Health Services Research in Dermatology, University Medical Center Hamburg-Eppendorf (UKE), Hamburg, Germany; 12 Department of Periodontics, Preventive and Restorative Dentistry, University Medical Center Hamburg-Eppendorf (UKE), Hamburg, Germany; 13 Department of Vascular Medicine, University Heart and Vascular Center, Hamburg, Germany; 14 Department of Otolaryngology, Head and Neck Surgery, Head and Neurocenter, University Medical Center Hamburg-Eppendorf (UKE), Hamburg, Germany; 15 Department of Oncology, Hematology, BMT with Section Pneumology, University Medical Center Hamburg-Eppendorf (UKE), Hamburg, Germany; 16 Institute for Sexual Research and Forensic Psychiatry, University Medical Center Hamburg-Eppendorf (UKE), Hamburg, Germany; 17 Institute for Systemic Neurosciences, University Medical Center Hamburg-Eppendorf (UKE), Hamburg, Germany; 18 Clinic of Neuroradiological Diagnostics and Intervention, University Medical Center Hamburg-Eppendorf (UKE), Hamburg, Germany; 19 Department of Psychiatry and Psychotherapy, University Medical Center Hamburg-Eppendorf (UKE), Hamburg, Germany; 20 Department of Neurology, University Medical Center Hamburg-Eppendorf (UKE), Hamburg, Germany; 21 Department for Cardiovascular Surgery, University Heart and Vascular Center, Hamburg, Germany; 22 Department of Oral and Maxillofacial Surgery, University Medical Center Hamburg-Eppendorf (UKE), Hamburg, Germany; 23 Prostate Cancer Center, Martini-Clinic, University Medical Center Hamburg-Eppendorf (UKE), Hamburg, Germany; 24 Department of Medical Psychology, University Medical Center Hamburg-Eppendorf (UKE), Hamburg, Germany; 25 Institute for Occupational and Maritime Medicine (ZfAM), University Medical Center Hamburg-Eppendorf (UKE), Hamburg, Germany; 26 Medical Clinic and Polyclinic III, University Medical Center Hamburg-Eppendorf (UKE), Hamburg, Germany; 27 Institute for Medical Sociology, University Medical Center Hamburg-Eppendorf (UKE), Hamburg, Germany; 28 Institute for Health Economics and Healthcare Research, University Medical Center Hamburg-Eppendorf (UKE), Hamburg, Germany; 29 Clinic of Ophthalmology, University Medical Center Hamburg-Eppendorf (UKE), Hamburg, Germany; 30 Institute of Human Genetics, University Medical Center Hamburg-Eppendorf (UKE), Hamburg, Germany; 31 Institute for Psychosomatic Medicine and Psychotherapy, University Medical Center Hamburg-Eppendorf (UKE), Hamburg, Germany; 32 Department of Electrophysiology, Hamburg University Heart Center, University Heart and Vascular Center, Hamburg, Germany; 33 Competence Center for Epidemiology and Health Services Research for Healthcare Professionals (CVcare), Institute for Health Services Research in Dermatology and Nursing (IVDP), University Medical Center Hamburg-Eppendorf (UKE), Hamburg, Germany; 34 Institute for Tumor Biology, University Medical Center Hamburg-Eppendorf (UKE), Hamburg, Germany; 35 Department of Pathology, University Medical Center Hamburg-Eppendorf (UKE), Hamburg, Germany; 36 Department of General Practice and Primary Care, University Medical Center Hamburg-Eppendorf (UKE), Hamburg, Germany; 37 Competence Center for Health Services Research in Dermatology (CVderm), Institute for Health Services Research in Dermatology and Nursing (IVDP), University Medical Center Hamburg-Eppendorf (UKE), Hamburg, Germany; 38 Airway Research Center North, German Center for Lung Research, Grosshansdorf, Germany

**Keywords:** Epidemiology, Prospective cohort study, Risk factors, Prevention, Coronary heart disease, Stroke, Dementia, Cancer, Health care, Vascular diseases, Oral health, Psychiatric and psychosomatic disorders, Ocular diseases, Respiratory diseases, Obesity, Nutrition, Lifestyle, Sexual dysfunction, Survivorship, Resilience, MRI imaging, Cardiac MRI, Brain MRI, Health service research, Hamburg

## Abstract

The Hamburg City Health Study (HCHS) is a large, prospective, long-term, population-based cohort study and a unique research platform and network to obtain substantial knowledge about several important risk and prognostic factors in major chronic diseases. A random sample of 45,000 participants between 45 and 74 years of age from the general population of Hamburg, Germany, are taking part in an extensive baseline assessment at one dedicated study center. Participants undergo 13 validated and 5 novel examinations primarily targeting major organ system function and structures including extensive imaging examinations. The protocol includes validate self-reports via questionnaires regarding lifestyle and environmental conditions, dietary habits, physical condition and activity, sexual dysfunction, professional life, psychosocial context and burden, quality of life, digital media use, occupational, medical and family history as well as healthcare utilization. The assessment is completed by genomic and proteomic characterization. Beyond the identification of classical risk factors for major chronic diseases and survivorship, the core intention is to gather valid prevalence and incidence, and to develop complex models predicting health outcomes based on a multitude of examination data, imaging, biomarker, psychosocial and behavioral assessments. Participants at risk for coronary artery disease, atrial fibrillation, heart failure, stroke and dementia are invited for a visit to conduct an additional MRI examination of either heart or brain. Endpoint assessment of the overall sample will be completed through repeated follow-up examinations and surveys as well as related individual routine data from involved health and pension insurances. The study is targeting the complex relationship between biologic and psychosocial risk and resilience factors, chronic disease, health care use, survivorship and health as well as favorable and bad prognosis within a unique, large-scale long-term assessment with the perspective of further examinations after 6 years in a representative European metropolitan population.

**Electronic supplementary material:**

The online version of this article (10.1007/s10654-019-00577-4) contains supplementary material, which is available to authorized users.

## Introduction

Within the last decades, a change in the disease pattern has been observed. Today, the majority of the ageing populations in industrialized parts of the world will survive acute events and move into survivorship living with one or more of the following conditions, e.g., cardiovascular and neurovascular disease, cancer, respiratory diseases, diabetes [1]. This change is mostly explained by better diagnostics leading to identification of disease at an earlier stage and to the development of more effective treatments of these diseases.

In addition to the change in disease patterns a change in diagnostic abilities and personalized treatment opportunities has occurred. The integration of imaging, molecular biology and clinical information holds promise to better detect at risk individuals and to personalize treatment.

Besides this new perspective of living for years at risk or in treatment for a chronic health condition, this may also influence physical, mental and sexual health, quality of life, general disability and society’s health expenses. In Germany, like in other affluent industrialized countries, the above mentioned chronic disease epidemic accounts for more than a third of the annual health expenditures, almost half of the total hospital payments [2]. It was estimated in 2013, that 500,000 potentially productive life years were lost due to premature death by these conditions in the working-age population (29–59 years of age [3]).

Observational studies have changed practice of medicine and lifestyle for millions of people [4], e.g., the Framingham Heart Study, which for the first time identified risk factors for cardiovascular events [4]. The conditions for those who actually survived these diseases were only a minor part of the agenda. Thus, we established a prospective observational cohort study that can address the impact of information from biological samples, medical examinations and imaging, classic self-reported questionnaire data and their interplay on our understanding of common disease development. Issues of disease development, pathophysiological understanding, artificial intelligence and survivorship constitute the cornerstones of the Hamburg City Health Study.

Moreover, we establish a biobank including a wide variety of biomaterials enabling molecular analyses, that—in total, will lead to a better understanding of health, disease and survivorship.

## Objectives

**Fig. 1:**
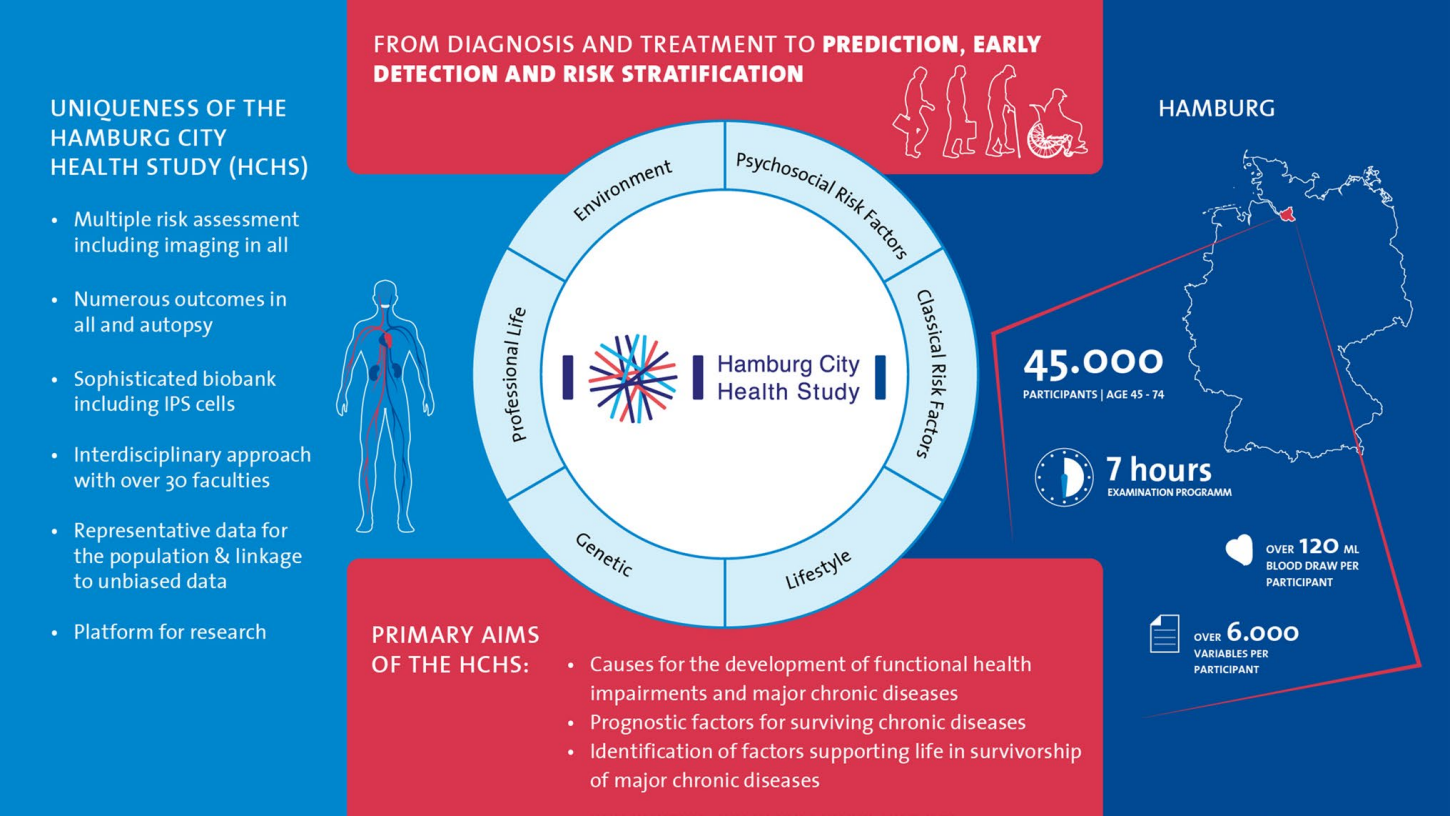
Summary of aims and uniqueness: the Hamburg City Health Study

The Hamburg City Health Study has established a unique research platform with multiple risk assessment, numerous outcomes and imaging examinations in all participants, a sophisticated biobank and interdisciplinary network to address a wide range of questions about more than 30 major chronic diseases (see Box 1 and Fig. 1) and survivorship.

**Box 1:** Overview of the main outcomes: the Hamburg City Health Study

Main outcome	
Coronary artery disease and myocardial infarction	
Atrial fibrillation	
Heart failure	
Dementia	
Stroke	
Cancer such as prostate cancer and skin cancer	
Chronic kidney diseases	
Arterial hypertension, hypercholesteremia and valvular diseases such as bicuspide aortic valve disease	
Migraine	
Musculoskeletal diseases such as osteoporosis and bone metastasis	
Ocular diseases such as glaucoma, macular degeneration, fundus hypertonicus, retinal vessel disease and neoplasm	
Oral health including periodontal disease and caries, oropharyngeal cancer and human papillomavirus (HPV)-infection	
Psychiatric and psychosomatic disorders such as mental disorder or late – last depression	
Pulmonary diseases such as chronic obstructive lung disease (COPD) and asthma	
Sexual dysfunction	
Skin diseases such as psoriasis, chronic wounds and inflammation	
Vascular diseases such as cerebrovascular disease, aortic aneurysm, thrombosis and peripheral arterial disease

Therefore, the primary aims of the HCHS are to investigate in detail:

- the causes for the development of functional health impairments and major chronic diseases,
- the prognostic factors for surviving chronic diseases and
- identification of factors supporting life in survivorship of major chronic diseases.

## Study design and methods

Hamburg is the second largest city in Germany with 1,830,584 million inhabitants (31.12.2017 [5] from all social classes living in 7 districts and 104 urban quarters. The city is mostly urban, but has also some rural areas and a large harbor contributing to the environmental exposures of the population. 839,389 persons are older than 45 years and in 2017 approximately 90,000 persons moved away. On an annual basis a sample census is carried out mapping social conditions of the German population including a random sample of 1% of the whole population. As shown in Box 2 the Hamburg population in comparison to the German population is characterized by 4% more other nationalities, people are 7% more often single, 14% have a higher education and a higher income.

**Box 2:** Selected characteristics of the target population: Results from the sample census (microcensus), Hamburg*

Feature	Category	Hamburg			Germany			*p* Value
		Overall	Men	Women	Overall	Male	Female	
		N(%)	N(%)	N(%)	N(%)	N(%)	N(%)	
*Age*								
	45–54	274.4 (42.9)	139.6 (44.3)	134.9 (41.5)	13,220 (39.8)	6657 (40.5)	6563 (39.0)	0.2615
	55–64	208.5 (32.6)	102.4 (32.5)	106.1 (32.7)	11,698 (35.2)	5798 (35.3)	5900 (35.1)	
	65–74	156.9 (24.5)	72.9 (23.2)	84.0 (25.8)	8335 (25.1)	3966 (24.2)	4369 (26.0)	
*Nationality*								
	German	1528 (84.4)	735 (82.9)	793 (85.8)	73,112 (88.5)	35,929 (87.6)	37,183 (89.4)	< 0.001
	Other	282 (15.6)	151 (17.1)	131 (14.2)	9526 (11.5)	5103 (12.4)	4423 (10.6)	
*Marital status*								
	Married	679 (37.9)	339 (38.7)	340 (37.1)	37,194 (45.0)	18,679 (45.5)	18,515 (44.5)	< 0.001
	Single	881 (49.1)	467 (53.2)	414 (45.1)	34,348 (41.6)	18,852 (45.9)	15,496 (37.2)	
	Divorced	134 (7.5)	53 (6.0)	81 (8.8)	5717 (6.9)	2415 (5.9)	3303 (7.9)	
	Widowed	99 (5.5)	18 (2.0)	82 (8.9)	5379 (6.5)	1087 (2.6)	4292 (10.3)	
School education**								
	Upper secondary education	721 (51.6)	362 (53.5)	359 (49.9)	22,543 (34.6)	11,757 (36.7)	10,786 (32.6)	< 0.001
	Lower secondary education	675 (48.4)	315 (46.5)	360 (50.1)	42523 (65.4)	20271 (63.3)	22253 (67.4)	
*Income in €*								
	500 < 899	75 (9.8)	25 (5.5)	50 (12.3)	10,256 (17.5)	3675 (11.9)	6581 (23.8)	< 0.001
	900 < 1299	126 (16.4)	49 (10.7)	76 (18.7)	11984 (20.5)	4662 (15.1)	7322 (26.5)	
	1300 < 1499	79 (10.3)	36 (7.9)	43 (10.6)	5834 (10.0)	2807 (9.1)	3027 (11.0)	
	1500 < 1699	87 (11.4)	42 (9.2)	45 (11.1)	5336 (9.1)	2818 (9.1)	2518 (9.1)	
	1700 < 1999	115 (15.0)	59 (12.9)	57 (14.0)	6538 (11.2)	3783 (12.2)	2755 (10.0)	
	2000 < 2599	69 (9.0)	95 (20.7)	73 (18.0)	8724 (14.9)	5654 (18.3)	3070 (11.1)	
	2600 < 3199	86 (11.2)	54 (11.8)	32 (7.9)	4146 (7.1)	2953 (9.5)	1193 (4.3)	
	3200 < 4499	76 (9.9)	55 (12.0)	20 (4.9)	3611 (6.2)	2788 (9.0)	822 (3.0)	
	≥ 4500	53 (6.9)	43 (9.4)	10 (2.5)	2170 (3.7)	1827 (5.9)	344 (1.2)	

*Data retrieved by the Mikrozensus 2016/2017 Statistisches Amt für Hamburg und Schleswig–Holstein. Absolut numbers in 1000 asked persons. The results of a 1% sample were extrapolated to the current population. The total number is varying due to missing data

**Based on the International Standard Classification of Education (ISCED) which belongs to the UNESCO

A total of 45,000 inhabitants, aged 45–74 years are to be included, identified by a random sample from the official inhabitant data file divided into six age and gender strata. The HCHS is a joint interdisciplinary endeavor of physicians and scientists from the University Medical Center Hamburg-Eppendorf. Over 30 departments and institutes from the University Medical Center Hamburg-Eppendorf work together in a unique cooperation at a single study center.

### Pilot study, timeline and examinations

From May 08, 2015 until January, 31 2016 1800 volunteers in the age group 18–85 were recruited by a commercial campaign in the leading newspaper from Hamburg and took part to validate the invitation process and train the study nurses in the examination procedures. Moreover, the manageability of the questionnaires was tested. This pilot study led to minor changes in these aspects. The first participant was enrolled on February 08, 2016 in the main study and the last participant is expected to be enrolled on November 30, 2022. The end of the first personal Follow-Up is planned 6 years later in 2028.

The participants are contacted by a letter to their home address containing the invitation and an information leaflet providing basic study information. Participants organize their own appointment at the epidemiological study center at the University Medical Center Hamburg-Eppendorf. The appointment is initiated by a study nurse explaining the study rationale and participants are asked to sign informed consent including study participation, an extraction of a skin punch to create induced pluripotent stem cells and either none, one or all of the following options: external, virtual or internal autopsy in the event of death. In the end, participants also sign a consent accepting that both double de-identified and pseudo-anonymized data may be transferred to cooperation partners. Participants are also asked for consent to match their health insurance and pension insurance data with the HCHS dataset. During a 7-h examination participants undergo validated examinations of different organ systems such as anthropometric measures, resting blood pressure measurements, ECG tracings as well as validated physical examinations. Novel parts include detailed cardiovascular, cognitive and oral health phenotyping, skin screening, pulmonary function test, muscle tests and optical coherence tomography (see Box 3 for an overview of all examinations). At the end of the visit, a letter is handed out containing results of all examinations and standardized recommendations to be followed by the participants. Following the discussion with the local ethical committee only clinically relevant results are provided to the participants. Before, during and after the baseline visit validated self-report questionnaires asking for life style and environmental conditions, dietary habits, quality of life, physical and sexual dysfunction, professional life, psychosocial context and burden, digital media use, medical and family history, occupational history as well as health care utilization are filled out (see Box 4 for an overview of all questionnaires).

**Box 3:** Overview of all components of the baseline examination high lightening novel and established clinical practice: the Hamburg City Health Study

Components of the baseline examination	Variables
*Novelty*	
2D and 3D transthoracic echocardiography	Complete screening of aortic-, mitral- and tricuspid-valve. 4D-echocardiography of every participant of the whole left and right heart. Measurement of ventricular and atrial strain.
Lung function	Performance of a broad/comprehensive screening by bodyplethysmography.
Ophthalmological examination	Assessment of the objective refraction and subjective visual acuity, imaging of the macular and papillary retinal layers including intravasal flow visualization using swept source optical coherence tomography.
Oral examination	Scaling of periodontitis severity according to the CDC-AAP criteria and whole mouth examination with assessment of caries (DMFT index) and tooth status.
Ultrasound of the abdominal aorta	Evaluation of the infrarenal abdominal aorta by using the b-mode and continuous-wave-Doppler-mode ultrasound to measure the diameter at the maximum, the outer-to-outer-method is used. Maximum flow-velocity is measured in the infrarenal aorta.
Ultrasounds of the peripheral arteries	B-mode and continuous-wave-Doppler-mode is used to evaluate flow-velocity and plaque burden of the common femoral, superficial femoral and popliteal arteries.
*Established clinical practice*	
2D and 3D transthoracic echocardiography	Volumes and function of all four heart chambers by 2D and 3D echocardiography; left ventricular diastolic function; left ventricular mass.
Ankle-brachial index (ABI) [7, 8]	Manually measurement (Doppler ultrasound) of systolic blood pressures at the posterior tibial, the anterior tibial, and the brachial arteries.
Anthropometry	Assessment of Weight, height and measurement of waist and hip circumferences.
Blood pressure measurement	Measurement after 5 min rest and 3 determination sitting and lying.
Cognitive function	Screening for impairment of cognitive function: Mini-Mental State Exam (MMSE), Clock Drawing Test (CDT); test of cognitive function: verbal fluency, semantic and vocabulary memory, visual attention and task switching, response inhibition, visuospatial abilities, cognitive processing speed, executive function.
Electrocardiography	Assessment with a standard digital 12-lead ECG and 2-min rhythm strip.
Lung function	Assessment of spirometry.
Muscle status and coordination	Assessment of hand-grip strength, Timed-up and go Test, Chair–Rising Test, Tandem Test.
Oral examination	Assessment of tooth status (DMFT index), pocket probing depths (periodontitis), bleeding on probing (gingivitis), dental plaque, condition of hard and soft tissues, assessment of craniomandibular dysfunctions, saliva and sulcus fluid sampling, oral hygiene behavior and oral health literacy.
Physical activity [9–11]	Objective measure of physical activity with Actigraph.
Standardized neurological examination	Assessment of the National Institutes of Health Stroke Scale.
Ultrasound of the carotid artery	B-mode and continuous-wave-Doppler-mode is used to evaluate flow-velocity and plaque burden of the carotid artery.
Ultrasound of the peripheral venous system	The b-mode is used for venous compression ultrasound at the femoral and popliteal vene.

**Box 4:** Overview of all components of the self-reported questionnaires: the Hamburg City Health Study

Component of questionnaire	
*Overview of dimensions and scales of the baseline and follow-up self-reported questionnaire*	
Adverse childhood experiences	Adverse childhood experiences questionnaire (ACE) [12, 13]
Alcohol use	Alcohol use disorders identification test (Audit-C) [14, 15]
Anxiety	Generalized anxiety disorder screener (GAD-7) [16]
Chronotype (single items)	Munich chronotype questionnaire (MCTQ) [17, 18]
Daytime sleepiness	Epsworth sleepiness skala (ESS) [19]
Depression severity	Patient health questionnaire (PHQ-9 D) [20–22]
Dermatology quality of life index	Dermatology life quality index (DLQI) [23]
Family history of disease	Physical & psychological diseases of parents
General practitioners (GP) guided care	Use of general practitioner
Health beliefs	Scale of the German health interview and examination survey for adults (DEGS-39) [24, 25]
General health perception	Scale of the German health interview and examination survey for adults (DEGS-29) [24, 25]
Health economics	Costs, EQ-5D-5L, ICER
Informal care	
Lifestyle medicine	European Prospective Investigation into Cancer and Nutrition (EPIC): Food Frequency [26] and Physical activity [27]
Living—environment—work	General housing data, technical data, quality of living, housing and work
Medical history	Allergies, gynaecology, skin diseases, cardiovascular diseases, cancer, ENT diseases, lung diseases, nephrology, neurology, orthopaedics, other diseases, dental and oral diseases
Medication-related beliefs	Beliefs about Medicines Questionnaire (BMQ Short form) [28, 29]
Migraine questionnaire	The rostock headache questionnaire (RoKoKo) [30]
Occupational exposure to fume, gases, organic or anorganic dust	Exposure assessment, specifically adapted for HCHS, based on questionnaire of the European community respiratory health survey (ECRHS), occupational module
Optimism, pessimism	SWOP
Oral health	Oral health literacy, oral hygiene, oral hygiene behaviour, oral health care utilization, fluoride prophylaxis, nutrition, dental service utilization [31–33]
Patient satisfaction	Zufriedenheit in der ambulanten Versorgung [Satisfaction in outpatient care] (ZAPA) [34]
Quality of life	European Quality of Life (EQ-5D-5L) [35], [36] Short-Form Health Survey (SF-8) [37, 38]
Resilience	RES-6
Risk factors and medical history	National cohort study (NAKO) [39]
Sexual dysfunctions	DSM—5 criteria
Shift and night work	career history, jobs with shift and night work [40, 41]
Social support	F-SozU-K14
Sociodemographic characteristics	i.e. Gender, age, nationality, mother language, religion
Somatic symptom	Patient Health Questionnaire (PHQ-15) PHQ-15 [42, 43]
Utilization of medical services	Scales of the German Health Interview and Examination Survey for Adults (DEGS-9, DEGS-10, ÖKO-DEGS-15) [24]
Significant life events	Social reactivity reaction scale (SRRS)
Smoking behavior	Fagerström questionnaire, smoking anamnesis
*Overview of dimensions and scales of the MRI self-reported questionnaire*	
Anxiety	Generalized anxiety disorder (GAD-2) [44]
Daily life	Social interaction, cognitive deficits, own psychological diseases & in the family
Depression	Patient health questionnaire (PHQ-9) [20, 21]
Emotional regulation	Questions from the response styles questionnaire (RSQ-D) [45, 46]
Emotional well-being	Current personal condition
Health advice	Scale of the German health interview and examination survey for adults (DEGS-11) [24]
Health behaviour measures	Scale of the German health interview and examination survey for adults (DEGS-20) [24]
Health care satisfaction	Zufriedenheit in der ambulanten Versorgung/[Satisfaction in outpatient care] (ZAPA) [34, 46]
Health literacy	Patient Activation Measure (PAM-13)
Health risks perception	
Media consumption	Patterns of media consumption
Medication-related beliefs	Beliefs about medicines questionnaire (BMQ Short form) [28, 29]
Personality	Eysenck personality questionnaire (EPG-RK) [47]
Quality of life	Short-Form Health Survey (SF-8) [37, 38]
Self-efficacy	Skala zur Allgemeinen Selbstwirksamkeits-erwartung [Self-efficacy scale] (SWE)
Significant life events	Social readjustment rating scale (SRRS)
Somatic Symptoms	Somatic symptom disorder-B criteria scale SSD-12) [48] Somatic Symptom Scale (SSS-8) [43]

Validated risk scores are used to identify individuals at risk for coronary artery disease, atrial fibrillation, heart failure, stroke and dementia. After the baseline investigation, these score-positive participants are invited to the imaging examination including an MRI—examination of the heart and the thoracic aorta and/or brain depending on the target disease [6]. In order to establish a general control group, 1500 random participants are invited to an MRI as well. Participants at risk for osteoporosis or suspected bicuspid aortic valve disease, prostate cancer, HPV-infection or dementia are recommended a further medical clarification.

### Laboratory parameters and biobanking

A panel of basic laboratory analyses are performed on the day of the visit in the study center. The assessed markers include: sodium, potassium, HbA1c, prostate specific antigen (PSA), creatinine, high sensitivity measured CRP, glucose, thyroid stimulating hormone (TSH), triglycerides, total cholesterol, HDL-cholesterol, LDL/HDL ratio, and N-terminal pro B-type natriuretic peptide (NTproBNP). Furthermore, a complete blood count is performed. Biomaterials used for biobanking include serum, plasma (EDTA, citrate), genomic deoxyribonucleic acid (DNA), ribonucleic acid (RNA) from whole blood and peripheral blood mononuclear cells (PBMCs), blood cells (erythrocytes, PBMCs), urine, saliva as well as tooth fluid and tonsils swabs. Additionally, from a random subset of study participants, skin stanza are collected of which fibroblasts are separated. These fibroblasts will be used for the generation of human induced pluripotent stem cells (hiPSCs) (for an overview of all biomaterials collected and planned measurements see Box 5). Subsequently, the biomaterial will be examined by state-of-the-art and innovative, high throughput approaches including analyses on the different OMICS levels such as genomics, transcriptomics, proteomics and lipidomics profiling. One part of the biobank is used for research projects within the first 6 years, the second part is stored for projects performed during the studies follow up and in the future.

**Box 5:** Overview of all collected biospecimen and purposes: the Hamburg City Health Study

Biospecimen	Novelty
Serum	Measurement of routine biomarkers as well as innovative markers such as non-coding RNA, proteomics, metabolomics and viruses
EDTA plasma	Measurement of routine biomarkers as well as innovative markers such as ncRNA, proteomics, metabolomics and viruses
Citrat plasma	Measurement of routine biomarkers and innovative markers of coagulation cascade
Genomic DNA	Measurement genomic and epigenomic markers
Whole blood RNA	Measurement of gene expression and expression of non-coding RNA
Erythrocytes	Measurement of innovative biomarkers and protein activity
PBMC	Generation of iPS cells, and cell culture experiments, Gene expression and measurement of circulating tumorcells (CTC)
Urine	Measurement of innovative markers
Saliva	Measurement of innovative biomarkers and oral microbiome
Tooth fluid	Measurement of oral microbiome
Tonsils swab	Measurement of oral microbiome and viruses
Skin Tissue	Generation of iPS cells

### Follow-up

Following the date of baseline examination, all participants will be contacted by mail containing a questionnaire which specifically ask participants to report any major medical event, medication, nutrition and lifestyle changes, physical and mental health, sexual dysfunction and overall quality of life as well as health care use. Participants are also asked to provide discharge letters or any kind of further information on their health such as diagnostic findings or images. This contact takes place on an annual basis for 5 years. An endpoint-committee will review all collected information for special endpoints. After 6 years all participants are invited to undergo the same examination and procedures as in the baseline visit. On a continuous basis the study center is in contact with public authorities and the cancer register about vital status, cause of death and cancer incidence and in contact with involved health and pension insurances to match with related individual routine data.

### Statistical analysis plan

The integrity of the collected data in the databases are controlled by detailed, predefined quality control algorithms according to standardized operation procedures (SOP) concerning detection of outliers, logically implausibility, or detect mistaken identity. Only quality controlled data will be used for statistical analyses.

In the analyses of baseline data methods for cross-sectional analyses will be applied. Univariate statistics for categorical variables will be presented as counts and proportions, and numeric variables will be presented as means, percentiles and standard deviations. Associations between baseline characteristics will be estimated by means generalized linear regression models.

For the full cohort as well as for sub-cohorts, time-to-event methods constitute the major approach for identifying and assessing risk factors for mortality and incidence or progression of diseases. Thus, when studying rates of a single event type, e.g., overall mortality, regression models for censored data will be used to simultaneously investigate the effects of risk factors of interest while adjusting for potential confounders.

In all analyses, effect estimates will be presented both, in relative terms as prevalence or risk ratios, and in absolute terms as means, rates or risks, the latter to assess public health implications. All effect estimates and statistical summaries will be presented with 95% confidence intervals, and, when appropriate, adjusted for multiple testing. For disease-specific mortality or endpoints other than death, competing risks will be accounted for. For repeated measurements in the second examination, regression models for longitudinal data will be employed. In all analyses observations may be correlated either in time (repeated measures) or between subjects (e.g. relatives). Appropriate statistical methods will be used to account for correlated observations, e.g. by means of mixed effect models, or in marginal models by means of generalized estimating equations (GEE). Special attention will be paid to potentially informative drop-out or failure to obtain further measurements due to the occurrence of a specific event. These issues will be addressed by joint models for longitudinal and time-to-event data. In cases of a substantial amount of missingness in dependent or independent variables, multiple imputation will be incorporated in the analysis, as a supplement to the complete case analysis. To ensure transparency and reproducibility of results, all statistical methods and codes will be described and stored centrally as a supplement to study protocols. This material will be available to other researchers upon request.

## Ethical concerns

During the study conception, the local ethics committee of the Landesärztekammer Hamburg (State of Hamburg Chamber of Medical Practitioners, PV5131) was consulted and its approval for the study protocol as well as the approval by the Data Protection Commissioner of the University Medical Center of the University Hamburg-Eppendorf and the Data Protection Commissioner of the Free and Hanseatic City of Hamburg were obtained. The study has been registered at ClinicalTrial.gov (NCT03934957). The procedures set out in this study, pertaining to the conduct, evaluation, and documentation, are designed to ensure that all persons involved in the study abide by Good Clinical Practice (GCP), Good Epidemiological Practice (GEP) and the ethical principles described in the current revision of the Declaration of Helsinki. The study will be carried out in keeping with local legal and regulatory requirements. The requirements of the GCP and GEP regulation will be adhered to. In order to be admitted to HCHS, all participants are to consent to participate only after the nature and scope of the study have been explained to and understood by them. Written informed consent is obtained from all participants. The examinations were chosen because of non-invasive nature of acquisition and standardized testing to assess intermediate phenotypes of the different diseases. Well-characterized intermediate phenotypes are of great value for screening, patient monitoring and in the context with clinical trials. At the end of the baseline- and MRI- examinations, all participants receive a standardized report of all relevant clinical results with a possible recommendation of referral to the general practitioner. Recommendations for all findings were defined before study start and standardized. Findings, which need clinical referral immediately, are defined as well and reported directly to the participants. If required, participants are also accompanied to the emergency department of the University Medical Center Hamburg-Eppendorf by a member of the study staff.

## Data protection concerns

During the study conception, the Data Protection Commissioner of the University Medical Center Hamburg-Eppendorf and the Data Protection Commissioner of the Free and Hanseatic City of Hamburg were consulted and their approval for the study protocol was obtained (D4/17.06-22). The requirements of the Federal Data Protection Law (Bundesdatenschutzgesetz), the European General Data Protection Regulation and the law of Hamburg Hospitals (Hamburger Krankenhausgesetz) will be adhered to. Subjects will be identified solely by means of an individual identification code and data are only collected, stored and analysed pseudo-anonymized. To enhance data confidentiality, data, lab samples and genetic results have different identification numbers. The identification numbers will change between data collection, storage and disclosure. To avoid mistakes in assigning the right identification number, the pseudo-anonymization is conducted electronically. All data will be stored at the study centre and all analytical activity will take place at dedicated workstations situated at the study centre. Extensive surveillance and login of all activity will be carried out.

## Strengths and limitations

Most importantly, the HCHS is a prospective population-based cohort study, which enables cause and effect analyses investigating major risk factors for a number of symptoms and chronic diseases. In addition, the extensive annual follow-up also contributes to the advantages of the HCHS. Further, the high number of examinations including a range of novel approaches in different disciplines contributes to the fact that data from the HCHS may unravel hitherto unknown causal associations. In line with this consideration, the extensive analyses planned with the use of biological samples giving basic genomic information in combination with phenotypic data will provide a rich data source for advanced analyses. The high number of participants secures that even relatively rare outcomes may be investigated. Furthermore, it is the ambition to link the data from the HCHS to socioeconomic information and individual records indicating information about diseases, prescribed medications, in- and outpatient health care use as well as sick leave etc. The cohort recruitment allows that prevalent cases of most chronic diseases will be enrolled and thereby opens up for early cross-sectional analyses investigating associations leading to hypothesis driven studies when the first 10.000 participants are included and when the cohort is complete. In line with this consideration, one may also point to the possibility of describing the distribution of diseases associated with patterns of several factors, i.e. socioeconomic status in diabetes patients, nutrition and lifestyle factors in dementia or the association between skin disorder and dental status. The design also offers an opportunity to conduct more complicated disease trajectory analyses using patterns of symptoms and diseases as both causes and effects. It is also of vital importance that this study offers an opportunity to investigate determinants of survival and survivorship in accordance with the aims of the study.

Even prospective cohort studies will have limitations due to selection bias in participation and recall bias. This study mostly relies on the recall of participants as the method of achieving information about central outcomes during the 6 years of follow-up. This limitation will be addressed by obtaining data from health insurance and pension funds. Compared to cohort studies investigating risk factors of diseases characterized by high incidence rates the waiting time for incident outcomes in diseases having low incidence rates is longer. Conducting a multidimensional study also reduces the number of participants with complete data, and as funding is limited, this problem represents a true limitation of the data set, which in the end will be less than a 100% complete. From a more technical point of view, some data may not be collected due to participants wish, lack of staff or technical malfunction of equipment. To address this limitation, standardized quality controls are performed weekly to detect missing data in order to ensure that no systematic processes are in function. Birthday, Christmas cards and annual newsletters will remind the participant to inform the study centre in the event of a medical endpoint. A number of standardized operating procedures for the recruitment, collecting and storage of data, quality control and analyses will be established. To avoid endpoint misclassification, an interdisciplinary endpoint committee will render an expert opinion on the basis of discharge letters or further information. The integrity of the collected data in the databases is controlled by detailed, predefined quality control algorithms according to standardized operating procedures in batches concerning detection of outliers, logically implausibility, or detect mistaken identity. Only quality-controlled data are used for statistical analyses.

## Conclusion

In future, data from HCHS will strongly contribute to our knowledge about risk factors for and prognostic factors in major chronic diseases, survival and survivorship. It will be a unique source due to the combination of self-reported data, detailed imaging data, a vast number of biological information and unbiased administrative data established independent of the hypothesis of the study. It is the aspiration that the inclusion of novel aspects in all exposure assessment methods concurrently with well-established, traditional epidemiological tools across all exposures and outcome will help in achieving information of such quality so that it can feed directly into public health policy with regard to prevention and survivorship related aspects. In line with this aspiration, the use of the data in real-life clinic is also one of the main intention in this study.

## Electronic supplementary material

Below is the link to the electronic supplementary material.

Supplementary material 1 (DOCX 105 kb)
