# Supplementary material for: Rationale and Design of the Hamburg City Health Study
Source: Eur J Epidemiol. 2019 Nov 8;35(2):169–81. doi: 10.1007/s10654-019-00577-4 (PMC7125064; doi:10.1007/s10654-019-00577-4)
Supplement: Supplementary file 1 — Supplementary material 1 (DOCX 105 kb) [file 10654_2019_577_MOESM1_ESM.docx]

**Supplement:**

***Founding Board:***

Adam, Gerhard

Blankenberg, Stefan (speaker)

Koch- Gromus, Uwe

Gerloff, Christian

Jagodzinski, Annika (assessor)

***List of Investigators:***

Adam, Gerhard

Aarabi, Ghazal

Augustin, Matthias

Behrendt, Christian

Beikler, Thomas

Betz, Christian

Blankenberg, Stefan

Bokemeyer, Carsten

Brassen, Stefanie

Brekenfeld, Caspar

Briken, Peer
Busch, Chia-Jung

Büchel, Christian

Debus, Eike Sebastian

Fiehler, Jens

Gallinat, Jürgen

Gellißen, Simone

Gerloff, Christian

Girdauskas, Evaldas

Gosau, Martin

Graefen, Markus

Hanning, Uta

Härter, Martin

Harth, Volker

Heydecke, Guido

Huber, Tobias

Jagodzinski, Annika

Johansen, Christoffer

Koch-Gromus, Uwe

Konnopka, Alexander

König, Hans-Helmut

Kromer, Robert

Kubisch, Christian

Kühn, Simone

Löwe, Bernd

Lund, Gunnar

Meyer, Christian

Nienhaus, Albert

Pantel, Klaus

Püschel, Klaus

Reichenspurner, Hermann,

Sauter, Guido

Scherer, Martin

Schiffner, Ulrich

Schnabel, Renate

Schulz, Holger

Smeets, Ralf

Spitzer, Martin S.

Terschüren, Claudia

Thomalla, Götz

Thederan, Imke

von dem Knesebeck, Olaf

Waschki, Benjamin

Wenzel, Jan-Peer

Wegscheider, Karl

Zeller, Tanja

Zyriax, Birgit-Christiane

***Steering Board:***

Augustin, Matthias

Blankenberg Stefan,

Gallinat, Jürgen

Gerloff, Christian

Härter, Martin

Jagodzinski, Annika

Johansen, Christoffer

Koch-Gromus, Uwe

Sauter, Guido

Zeller, Tanja

Wegscheider, Karl

Betz, Christian/ Heydecke, Guido/ Gosau, Martin

***Research consortium:***

Aarabi, Ghazal

Andrees, Valerie

Behrendt, Christian Brassen, Stefanie

Brekenfeld, Caspar

Brünahl, Christian

Busch, Chia-Jung

Freitag, Janina

Gallinat, Jürgen

Gellißen, Susanne

Girdauskas, Evaldas

Heidemann, Christoph

Hussein, Yassin

Klein, Verena

Kofahl, Christopher

Kohlmann, Sebastian

Konnopka, Alexander

Kühn, Simone

Lühmann, Dagmar

Lund, Gunnar

Nagel, Lina

Magnussen, Christina

Meyer, Christian

Petersen, Elina

Scherschel, Katharina

Schiffner, Ulrich

Schnabel, Renate

Schulz, Holger

Seedorf, Udo

Smeets, Ralf

Thederan, Imke

Terschüren, Claudia

Thomalla, Götz

Waschki, Benjamin

Zeller, Tanja

Zyriax, Birgit-Christiane
